# Supplementary figures and images for: Mice lacking the mitochondrial exonuclease MGME1 develop inflammatory kidney disease with glomerular dysfunction
Source: PLoS Genet. 2022 May 9;18(5):e1010190. doi: 10.1371/journal.pgen.1010190 (PMC9119528; doi:10.1371/journal.pgen.1010190)

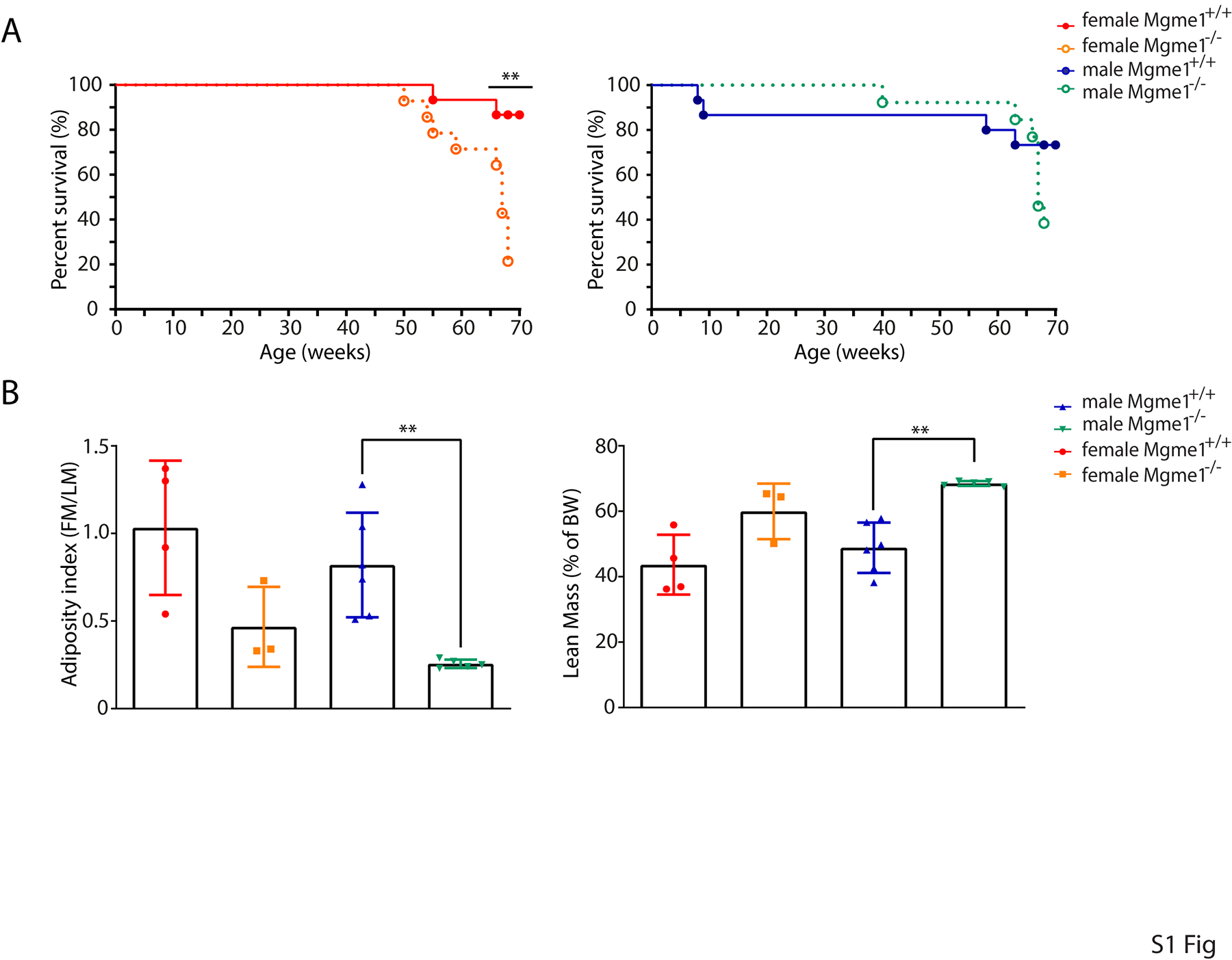

Supplement: S1 Fig — (A) Survival percent by sex. The attrition rate in females was significant with a p = 0,0017 Log-rank (Mantel-Cox) test. 11 from 14 female mutants and 2 from 15 control, 8 from 13 males mutants and 4 from 15 control animals did not reach the end of phenotyping. (B) Lean mass indicated as percent of body weight and adiposity index intended as ratio between fat and lean mass of 68 week-old n = 4 wild-type and 3 Mgme1-/- females and 6 wild-type and 5 Mgme1-/- males. Fat mass is indicated as percent of bodyweight. Values are given as mean ± SD, ** P ≤ 0.01, Unpaired Mann Whitney test. (TIF) [file pgen.1010190.s001.tif]

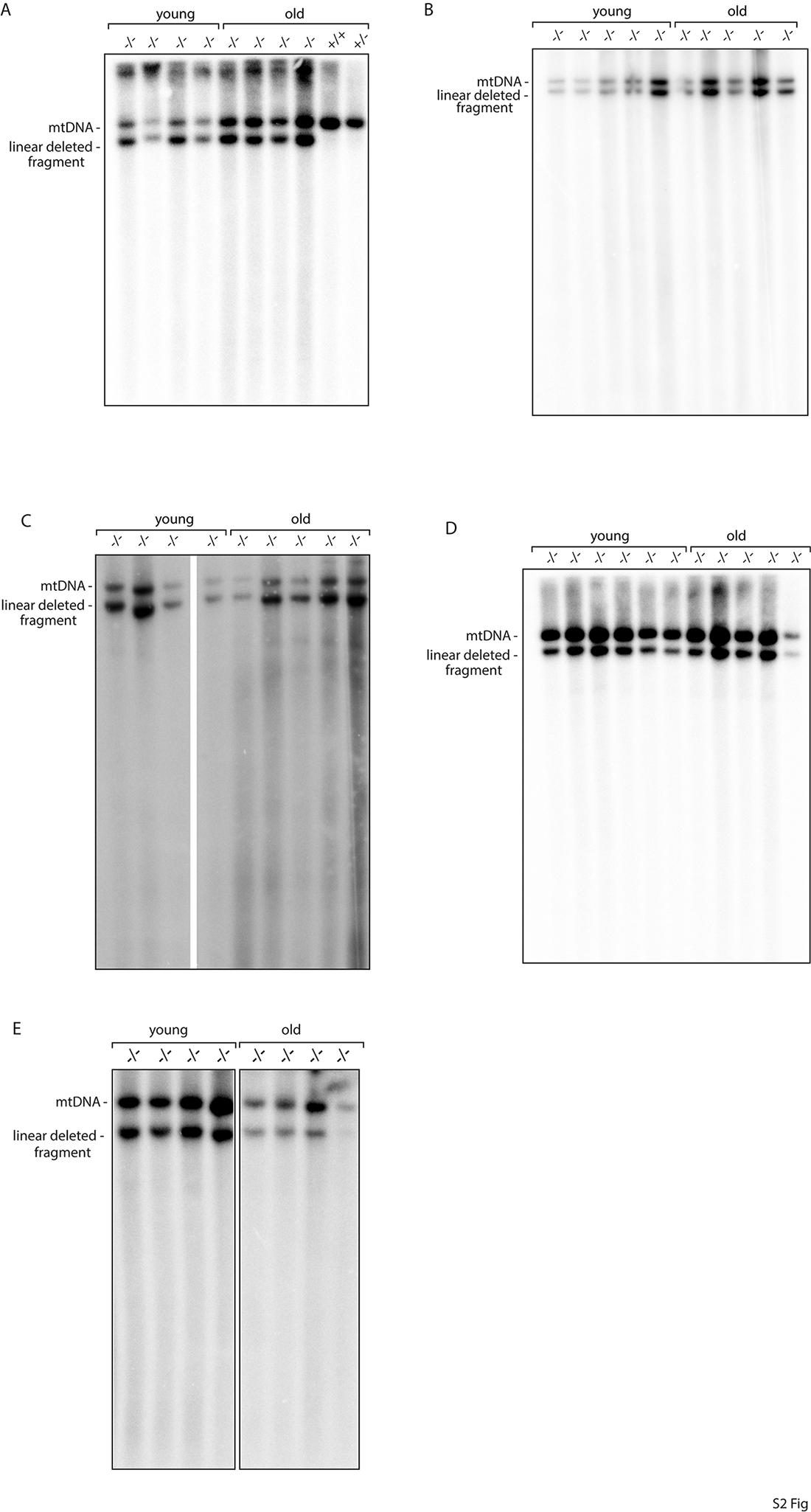

Supplement: S2 Fig — (A) liver, (B) kidney, (C) heart, (D) skeletal muscle and (E) brain. (TIF) [file pgen.1010190.s002.tif]

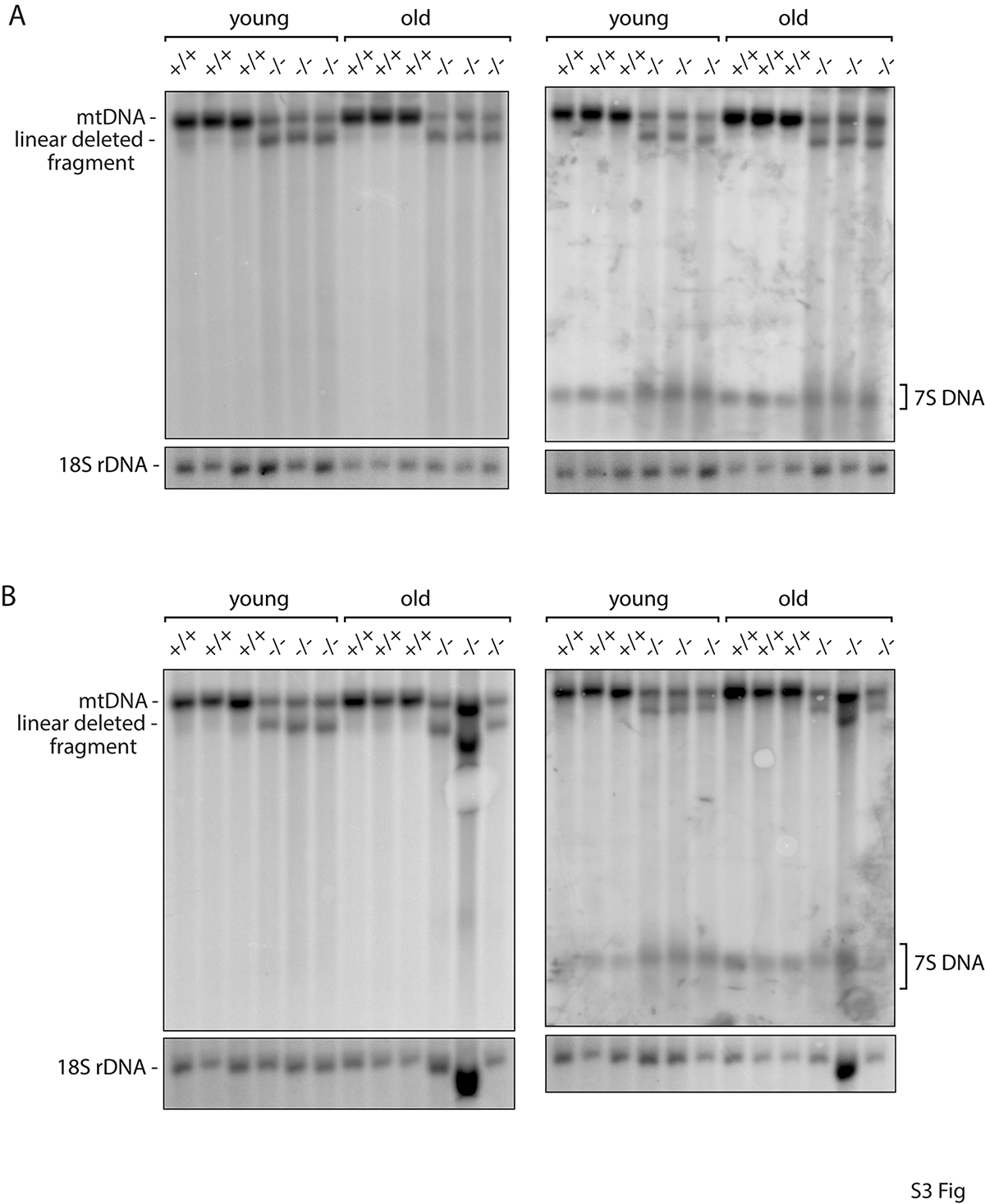

Supplement: S3 Fig — (A) heart (B) kidney. Panels on the left were used for quantification of total and full length mtDNA levels and the panels on the right for quantification of 7S DNA. The 18S rDNA was used as a loading control. (TIF) [file pgen.1010190.s003.tif]

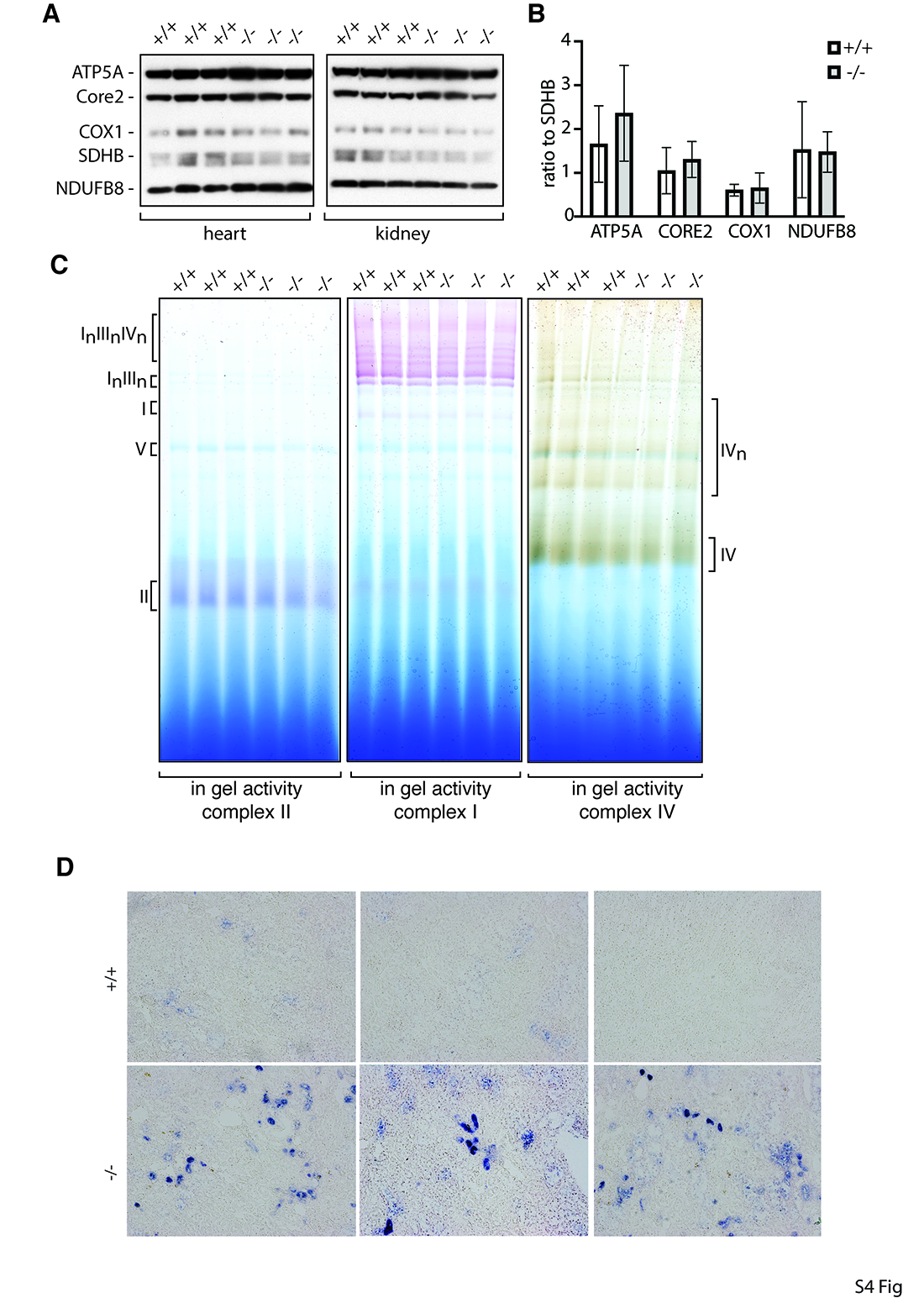

Supplement: S4 Fig — (A) Steady-state levels of OXPHOS subunits in heart and kidney of control (+/+) and Mgme1 knockout (-/-) mice. (B) Quantification of steady state levels of OXPHOS subunits from kidney tissue. (C) BN-PAGE analysis followed by in-gel enzyme activities of complexes I, IV and II (loading control) in Mgme1 knockout (-/-) and wild-type (+/+) kidney mitochondria at 55 weeks of age. (D) NBTx staining of kidney sections from Mgme1 knockout (-/-) and wild-type (+/+) mice. Representative pictures from multiple analysed sections from 5 wild-type (+/+) and 5 knockout (-/-) animals are shown. (TIF) [file pgen.1010190.s004.tif]
