# Supplementary material for: Mice lacking the mitochondrial exonuclease MGME1 develop inflammatory kidney disease with glomerular dysfunction
Source: PLoS Genet. 2022 May 9;18(5):e1010190. doi: 10.1371/journal.pgen.1010190 (PMC9119528; doi:10.1371/journal.pgen.1010190)
Supplement: S1 Table — In orange: Modified protocol for the Mgme1 knockout mice and controls. (PDF) [file pgen.1010190.s005.pdf]

S1 Table

		Age [weeks]															
Module	Methods	8	...	68	...	71	72	73	74	75	76	77	78	79	80	81
General	Age Monitoring															
Behaviour	Open field															
	Acoustic startle response & PPI															
Neurology	Modified SHIRPA, grip strength															
Clinical Chemistry	Clinical Chemistry after fasting															
Nociception	Hot plate															
Dysmorphology	Anatomical observation															
Allergy	Transepidermal water loss (TEWL)/ Body surface temperature															
Energy Metabolism	Indirect calorimetry, NMR															
Clinical Chemistry	IpGTT															
Cardiovascular	Awake ECG / Echocardiography															
Eye function	Scheimpflug imaging, OCT, LIB, drum															
Neurology	ABR (Auditory brain stem response)															
Dysmorphology	X-ray															
Energy Metabolism	NMR															
Clinical Chemistry	Clinical Chemical analysis, hematology															
Immunology	Flow cytometry, Immunoglobulin isotyping															
Allergy	MSD Panel 3 (IgE, IL6, TNF, insulin concentration), Cytokines															
Molecular Phenotyping (optional)	Expression profiling															
Pathology	Macro & microscopic analysis															
