# Supplementary material for: Mice lacking the mitochondrial exonuclease MGME1 develop inflammatory kidney disease with glomerular dysfunction
Source: PLoS Genet. 2022 May 9;18(5):e1010190. doi: 10.1371/journal.pgen.1010190 (PMC9119528; doi:10.1371/journal.pgen.1010190)
Supplement: S2 Table — (PDF) [file pgen.1010190.s006.pdf]

S2 Table

Results of the Clinical Chemistry Screening *(Wilcoxon Rank Sum Test, age of the mice: 68 wks)*

Mgme1	**female**		**male**		**female**	**male**	**overall**
	wt	hom	wt	hom			
	n=4	n=3	n=6	n=5			
	median [25%, 75%]	median [25%, 75%]	median [25%, 75%]	median [25%, 75%]	p-value	p-value	p-value
**Sodium [mmol/l]**	136 [135 , 138]	139 [138 , 144]	142 [140 , 143]	146 [144 , 149]	0.229	0.082	0.04
**Potassium [mmol/l]**	3.9 [3.6 , 4]	4.1 [3.9 , 4.5]	4.1 [4 , 4.2]	4.9 [4.6 , 5]	0.629	0.004	0.014
**Chloride [mmol/l]**	119.4 [118.2 , 120.2]	116.9 [116.9 , 119]	118.7 [117.7 , 120]	115 [111.8 , 115.1]	0.8	0.004	0.008
**Total protein [g/l]**	57.5 [56.8 , 58.5]	54.7 [47.2 , 58]	56.6 [54.2 , 57.5]	55.6 [55.6 , 56.1]	0.629	0.894	0.395
**Albumin [g/l]**	27.9 [26.9 , 28.4]	24.9 [20.4 , 26.4]	27.1 [26.5 , 28.5]	25.2 [24.9 , 25.4]	0.229	0.177	0.036
**Creatinine enz. [µmol/l]**	8.8 [8.37 , 9.26]	26.52 [17.24 , 27.23]	9.98 [8.27 , 10.38]	26.7 [23.34 , 51.1]	0.4	0.004	0.002
**Urea [mmol/l]**	9.38 [9.19 , 9.61]	25.92 [18.07 , 34.37]	9.18 [8.96 , 10.03]	38.13 [34.7 , 54.81]	0.057	0.004	< 0.001

Mgme1	**female**		**male**		**female**	**male**	**overall**
	wt	hom	wt	hom			
	n=4	n=3	n=6	n=5			
	median [25%, 75%]	median [25%, 75%]	median [25%, 75%]	median [25%, 75%]	p-value	p-value	p-value
**Calcium [mmol/l]**	2.32 [2.31 , 2.33]	2.64 [2.48 , 2.7]	2.36 [2.34 , 2.39]	2.82 [2.6 , 2.95]	0.286	0.002	0.003

**Inorganic phosphate [mmol/l]**	2.17 [2.12 , 2.29]	2.39 [2.29 , 2.44]	2.18 [1.92 , 2.48]	2.06 [1.93 , 2.54]	0.629	0.792	0.829
**ALP [U/l]**	106 [102 , 116]	171 [151 , 266]	68 [62 , 70]	198 [153 , 343]	0.114	0.004	0.001
**Iron [µmol/l]**	15.543 [14.876 , 16.532]	13.967 [13.143 , 15.364]	19.204 [16.823 , 21.585]	13.465 [9.956 , 14.468]	0.4	0.03	0.007
**Lactat (AU400) [mmol/l]**	5.18 [5.04 , 5.46]	7.39 [7.22 , 9.12]	8.45 [8.3 , 9.04]	6.92 [6.35 , 8.25]	0.057	0.177	0.696
**Unsaturated iron binding capacity [µmol/l]**	63.1 [60.9 , 65.2]	52 [39.5 , 56.4]	62.1 [60 , 63.1]	49.5 [46.9 , 53.2]	0.114	0.082	0.004
**Total iron binding capacity [µmol/l]**	79.3 [77.9 , 80.2]	64.3 [52.7 , 70.9]	79.2 [76.7 , 80.9]	63 [61.4 , 65.9]	0.114	0.017	0.001
**Calc transferrin saturation [%]**	19.9 [19.2 , 20.8]	21.6 [20.4 , 27.8]	24.2 [21.2 , 29]	21.4 [16 , 21.4]	0.629	0.16	0.617
Mgme1	**female**		**male**		**female**	**male**	**overall**
	wt	hom	wt	hom			
	n=4	n=3	n=6	n=5			
	median [25%, 75%]	median [25%, 75%]	median [25%, 75%]	median [25%, 75%]	p-value	p-value	p-value
**Cholesterol [mmol/l]**	2.065 [1.944 , 2.169]	4.942 [3.478 , 5.947]	2.159 [1.921 , 2.39]	6.506 [4.942 , 6.734]	0.229	0.004	0.001
**Triglyceride [mmol/l]**	0.677 [0.664 , 0.751]	0.544 [0.524 , 0.947]	0.633 [0.592 , 0.783]	1.363 [0.96 , 1.989]	0.629	0.009	0.083
**ALAT/GPT [U/l]**	82 [60 , 97]	29 [28 , 52]	58 [48 , 85]	24 [23 , 45]	0.229	0.093	0.022
**ASAT/GOT [U/l]**	122 [105 , 143]	111 [98 , 169]	172 [122 , 213]	94 [89 , 94]	1	0.009	0.025
**alpha-Amylase (CNPG3) [U/l]**	712.6 [656.53 , 746.37]	1131.9 [947.44 , 1199.61]	819 [788.59 , 841.08]	1078.74 [1034.83 , 1127.19]	0.114	0.004	0.001
**Glucose [mmol/l]**	15.62 [15.36 , 15.92]	14.2 [10.91 , 16.66]	13.55 [11.72 , 14.53]	9.46 [8.24 , 14.64]	0.629	0.329	0.173

**LDH [U/l]**	603.3 [546.2 , 730.4]	344.3 [334.4 , 686.4]	723.1 [589.4 , 840]	450.6 [348.7 , 524.5]	0.629	0.03	0.043
