# Supplementary material for: Mice lacking the mitochondrial exonuclease MGME1 develop inflammatory kidney disease with glomerular dysfunction
Source: PLoS Genet. 2022 May 9;18(5):e1010190. doi: 10.1371/journal.pgen.1010190 (PMC9119528; doi:10.1371/journal.pgen.1010190)
Supplement: Attachment — Submitted filename: Response Milenkovic 01.03.2022.docx [file pgen.1010190.s008.docx]

**Comments to reviewers, Milenkovic et al. (PGENETICS-D-21-01644)**

Reviewer #1

The manuscript by Milenkovic et al. characterizes the aging phenotype of mice lacking MGME1 and shows that these mice present with body weight loss, retinopathy, a kidney inflammation consistent with nephrotic syndrome, leading to premature death. The same group has previously shown that young mice lacking MGME1 show abnormal mtDNA replication, that leads to accumulation of linear mtDNA fragments (in manuscript citation [13]). In this manuscript, comparison of the relative content of linear mtDNA fragments between young (10 weeks) and old (50 weeks) animals showed that MGME1 is not necessary for the degradation of the linear mtDNA fragments, as no accumulation during aging was detected. To prove, that linear mtDNA fragments are indeed degraded in MGME1-lacking mice, in organello pulse-chase experiment in isolated heart mitochondria was performed. The manuscript is well written and data are clearly presented.
Questions:
Was there a decrease in the mtDNA levels in aged animals compared to the younger ones?
Response:

We present new Southern blot analyses performed on total DNA isolated from heart and kidney of wild-type and *MGME1*^-/-^ mice. Our data show that the amounts of total mtDNA (full-length mtDNA + linear deleted mtDNA) and full-length mtDNA are significantly decreased in knockout animals (Fig 2C and D, Fig S3 A and B). However, we do not see any significant age-associated effect on the degree of mtDNA depletion (Fig 2C and D, Fig S3 A and B).

Was there an accumulation of the 7S DNA in the aged mice compared to younger mice? Determination of 7S DNA levels in aged vs. young mice would by the same logic as for the linear mtDNA fragments show if MGME1 is responsible for the degradation of this DNA, as it was suggested previously?

Response:

We have performed new Southern blot analyses to assess levels of 7S DNA in heart and kidney of young and old *MGME1*^-/-^ mice and corresponding controls (Fig 2C and D, Fig S3 A and B). We found no effect on 7S DNA accumulation with age, which is consistent with previously published data. MGME1 is known to process the 5`end of 7S DNA, which leads to increase of its stability (Kornblum et al., 2013; Nicholls et al., 2014; Matic et al., 2018). Moreover, lack of MGME1 also severely impairs *de novo* synthesis of 7S DNA (Matic et al., 2018). These findings suggest that decreased synthesis of 7S DNA is counteracted by its increased stability in the absence of MGME1, consistent with the findings in this manuscript. In further support of this model, it has been reported that overexpression of wild-type or nuclease-inactive MGME1 in human cell lines leads to depletion of 7S DNA (Szeczesny et al., 2013).

There is an increase in the total amount of newly synthetized mtDNA in MGME1-/- mice in the in organello pulse/chase experiment, which seems to be rapidly degraded during the chase period. Is there a faster degradation of the newly synthetized mtDNA in MGME1-/- mice compared to controls and does this lead to the mtDNA depletion, rather than a problem with replication?

Response:

We and others (Kornblum et al., 2013; Nicholls et al., 2014; Matic et al., 2018) have previously published that MGME1 deficiency leads to a multitude of replication stresses, i.e. accumulation of 7S DNA, replication stalling and accumulation of substantial amounts of linear deleted fragments (that are products of nonproductive replication). A robust comparison of the rate of degradation of newly synthesized mtDNA is difficult because of the mtDNA depletion in the MGME1^-/-^ mitochondria in comparison with control mitochondria. The conclusion that MGME1 is not necessary for degradation of linear deleted mtDNA fragments is supported by the observed rapid degradation of linear deleted mtDNA in mitochondria lacking MGME1.

Were any inflammatory factors (increase in IL6/TNF/cytokines) detected in the aged mice, as Table S2 indicates that Immunology and Allergy module was performed?

Response:

We present new measurements showing age-associated increases of several proinflammatory cytokines in plasma of *MGME1*^-/-^ animals (new Fig. 4), consistent with the age-associated inflammatory kidney phenotype in these animals.

Minor comments:

- Please use decimal point in P-values in the paragraph “MGME1 deficiency causes kidney inflammation and nephropathy”

Response:

We have corrected this error in the revised version of the manuscript.

- Abstract: “formation of a linear deleted mtDNA fragments” – delete “a”

Response:

We have corrected this typo in the revised version of the manuscript.

Reviewer #2

 This manuscript by Milenkovic et al, expands upon the understanding of the role of the mitochondrial genome maintenance exonuclease (MGME1) in vivo. In a previous study the authors examined the role of MGME1 at early ages, but this study utilized aged knockout mice to analyze the function of MGME1 and the associated disease pathophysiology, as MGME1 mutations generally manifest as disease later in life in patients.

While aging the MGME1 knockouts it was found that these animals died prematurely, so doing the standard late-adult phenotyping (at 71 weeks) was not possible. Instead, the extensive phenotyping analyses were performed at 50 weeks. This modified “late-adult” phenotyping protocol also included neurological and morphological examinations, cardiovascular monitoring, and body composition measurements to give a complete and extensive assessment of the model. From these studies the authors concluded that striking phenotypes of the aged MGME1 knockout mice included reduced weight gain during aging and later a marked weight loss. Additionally, the MGME1 knockout mice developed cataracts due to abnormal fiber rearrangement and infiltrating epithelial cells in the lens. Retinopathy as also identified, where the overall thickness of the retina and retinal layers was reduced even though all the layers of the retina were present. Renal failure was observed through elevated plasma urea and creatinine levels. The nephrotic syndrome associated with renal failure was further confirmed through histological assessments of the kidney glomeruli. Furthermore, there was detection of infiltrating T and B cells in the kidneys.

This conclusions from this manuscript are novel and compelling for the mitochondrial biology field and the overall understanding of how defective mtDNA replication can lead to manifestations of inflammatory disease. The authors show evidence that contradicts the previous proposal where MGME1 plays a role in the degradation of linear mtDNA, and instead assert that MGME1 plays a crucial role in the completion of mtDNA replication. From the phenotyping experiments the authors assert that the loss of MGME1 leads to a dramatic phenotype that includes progressive weight loss, retinopathy, cataracts, and nephrotic disease. However, there are some lingering questions that arise, particularly relating to how the MGME1 knockout mouse compares to other models of mitochondrial DNA instability or defective replication that could be discussed in the text.

1) One of the major conclusions the authors draw from their studies is that MGME1 does not play a role in degrading linear mtDNA fragments, as proposed by Peeva et al (2018). However, that paper and the papers by Medeiros et al (2018) and Nissanka et al (2018), also show that TWNK and POLG can also play a role in degrading linear mtDNA fragments. Can the authors comments on why they think these other proteins do not compensate for the loss of MGME1 to degrade linear mtDNA?

Response:

MGME1 has a strong preference for degrading single-stranded DNA species in the 5′-3′ direction (Kornblum et al., 2013; Szeczesny et al., 2013). The studies mentioned by the reviewer suggest that the POLG 3´-5´exonuclease activity and the TWNK DNA unwinding activity are needed for the degradation of linear mtDNA fragments. It is difficult to see how the exonuclease domain of POLG (3′-5′ degradation) or the helicase TWNK (DNA-unwinding) can compensate for the proposed role for MGME1 in DNA degradation. In fact, the most straightforward interpretation of our data is that MGME1 is not required for degradation of linear deleted mtDNA.

2) In Figure 2 the authors show that the levels of the linear deletion are not significantly altered between young and aged MGME1 knockout animals in different tissues. Are the levels of total mitochondrial DNA altered between young and aged MGME1 knockout animals?

Response:

Please see response to Reviewer 1 and new Fig 2 and Fig S3

3) With the conclusion that the impaired mitochondrial DNA replication is directly responsible for the kidney inflammation, could to authors comment on how their studies with the MGME1 knockout mouse compare to other mouse models of impaired mitochondrial DNA replication?

Response:

Unfortunately, it is not possible to directly compare MGME1 knockout mice with knockouts of other replication factors. The basal mtDNA replication components (mtSSB, Twinkle, PolgA) are essential for mouse embryonic development and whole body knockout mouse models are thus unavailable. To our knowledge, there are no published kidney-specific knockout models of the mtDNA replication-related proteins. The mtDNA mutator mice, i.e. knock-in mice expressing proofreading-defective mitochondrial DNA polymerase, also accumulate linear deleted molecules. However, the mtDNA mutator mice additionally have drastically elevated levels of mtDNA point mutations and develop progeria with complex phenotypes from multiple affected organs. Kidney inflammation has not been reported in these animals. Mouse models with ablated mitochondrial transcription factor A (TFAM) expression in tubule cells (Chung et al., 2019) and other epithelial cells of kidney (Ishi et al., 2020) have renal inflammation with fibrosis or severe cystic kidney disease, respectively. Both of these deficiencies ultimately result in renal failure.

4) One of the hallmarks of the MGME1 knockout mouse model is the formation of the linear mtDNA deletion, which is also seen in the POLG mutator mouse. Comparatively however, the POLG mutator mouse does not have the same effect on mtDNA replication as the MGME1 knockout. Can the authors discuss on if they think that these linear deletions cause the systemic inflammation that causes the progressive kidney disease only in the MGME1 knockouts compared to the POLG mutator?

Response:

The accumulation of the linear subgenomic mtDNA is common molecular feature of the MGME1 knockout mouse model and the mtDNA mutator mice. However, the amount of linear deleted mtDNA in the MGME1-deficient animals is almost double in comparison with the mtDNA mutator mice (Matic et al., 2018). Furthermore, the mtDNA mutator mouse have drastically elevated levels of mtDNA point mutations and develop progeria with complex phenotypes from multiple affected organs. It is possible that the lower amount of linear deleted mtDNA and the shorter life span explains why the mtDNA mutator mouse does not develop kidney inflammation at the end of life.

Reviewer #3:

Mice Lacking the Mitochondrial Exonuclease MGME1 Develop Inflammatory Kidney Disease with Glomerular Dysfunction
Summary: The current publication reports on the age-associated pathology in Mgme1 KO mice and aims to expand on the role of MGME1.
The most impressive part of this manuscript is the amount of detail given to the mouse studies. It is evident that the parameters to arrive at these observational findings were meticulously planned, and there are vast amounts of data that were collected from these animals. The authors focused their attention on some of the striking pathophysiological features of these mice. Namely: survival, weight loss, body mass composition, blood chemistry and gross histology. In addition, they tackle the ongoing controversy in the field regarding the role of MGME1 in degrading linear deleted mtDNA.

Regarding the observational aspect of this publication the following points may be worth noting:
• In figure 3, where the author reports the gross eye histological images, a close-up section of the WT lens isn’t provided to have a full comparison to a KO counterpart. At the same time, the author signals that there is an invagination visible in the KO that to the untrained eye looks like tissue processing artifact. This same artifact can be seen in pane A just right of center towards the top of the image. Is this also a tissue invagination? Are there more in the KO? If perhaps it is an artifact perhaps highlighting may not serve to highlight the differences between WT and KO. The differences in the lens epithelium are evident enough. And if the differences lie in quantities, then this should be stated.

Response:

Below, we provide an example of a close-up image of a wild-type (+/+) lens (panels A). There is no invagination of the lens capsule as it is continuous and smooth without any evident atypical structure. In contrast, the *MGME1*^-/-^ lens capsule (-/-; panels B), show an invagination seen as a growing-in from the capsule material between the epithelial cells. We are confident that this change is morphological. We routinely use embedding of the eye in plastic as it very well preserves morphological features. The invagination changes are present only in the lens of the *MGME1*^-/-^ and not in control animals.

• In figure 1 and supplemental figure 1, there is a decrease in fat mass (%BW) and conversely there is an increase in lean mass (%BW). This is interesting considering MGME1 human pathology where muscle wasting has been reported. Although logically, both muscles and fat must be wasting in these animals, perhaps a mention or presentation of a graphic showing the decrease in lean muscle mass across time can further provide support for how this model is like human disease, as is the presented, it shows an increase in lean muscle mass (as a percentage of total body weight).

Response:

The point concerning the lean mass can unfortunately not be addressed as we do not have time course data. Usually, we perform NMR measurement analyses at two time points (5 weeks apart) to monitor possible body composition changes with time. As we had to alter our plans due to the health of the animals, a second measurement was not possible to perform. The observed body weight decline seems to be mainly explained by fat reduction in the time window we measured. The parameter lean mass as obtained from NMR analysis is not only muscle mass but also includes other tissues, except fat and body fluids. We can therefore not give a clear statement about the muscle mass.

• In supplemental figure 2, the author reports that there is no apparent COX deficiency or OXPHOS deficiency in Mgme1 KO animals, however only the blot and BN-PAGE gel is shown. Consider quantifying these data and drawing statistics to show this very same conclusion as it is a subsection in the manuscript and not a point in one of the sections. Additionally, consider mentioning how the NBTx staining of the kidney sections showing COX deficient cells fits into the greater picture of kidney physiology. Perhaps, determining the identity of these cells (if possible) may provide a clearer picture of how kidney disease comes about and an explanation to what appears to be incongruous findings.

Response:

We have now performed quantification of the steady-state levels of various OXPHOS subunits in kidneys of old wild-type and control animals (Fig. S4B). The conclusion remains the same, i.e. the steady-state levels of OXPHOS subunits are not affected by MGME1 deficiency.

Additionally, consider mentioning how the NBTx staining of the kidney sections showing COX deficient cells fits into the greater picture of kidney physiology. Perhaps, determining the identity of these cells (if possible) may provide a clearer picture of how kidney disease comes about and an explanation to what appears to be incongruous findings.

Response:

It is unfortunately difficult to assess the cell types that are OXPHOS deficient on NBTx staining of the kidney in mice lacking MGME1. In addition, we know from previous mouse work that OXPHOS deficiency often preceedes cell loss. Therefore, this question is best addressed by following the progression of OXPHOS deficiency in kidneys of MGME1-deficient mice at different ages. Protocols will also have to be developed to combine NBTx staining with immunofluorescence or in situ hybridization to robustly identify affected cell types in the kidney. Given the experimental complexity and the time required to breed and age new MGME1 knockout cohorts, we prefer to address this question in a separate study.

When it comes to the experimental aspect of the manuscript, the author claims that the findings in this manuscript contradict a previous publication that states that MGME1 degrades long linear mtDNA molecules. In the current manuscript, Mgme1 KO animals are associated with de novo formation of linear fragments that are constantly made and degraded. This is only partially substantiated by the experimental data presented in figure 2. Not only does the pulse chase experiment have no signal for the WT samples, but there is no control for what typical linear fragment degradation (in WT cells) would look like in this experiment. Although WT cells have no linear deleted mtDNA, the author states that the degradation of linear fragments in these Mgme1 KO mice is not affected by the absence of MGME1. What if the absence only dampens degradation without full ablation? Without a comparative linear deleted fragment degradation example in WT mice this conclusion cannot be drawn. What can be gathered is that the linear deleted fragment is degraded, and that perhaps there is greater mtDNA turnover in the Mgme1 KO cells which may lead to production of this fragment. If possible, providing more experimental data about mtDNA turnover in this set of mice 1) may demonstrate the differences in mtDNA quantity between the two groups and 2) may elucidate whether the production of these linear mtDNA fragments is directly tied to mtDNA replication. An interesting point seeing as MGME1 is involved in post replication processing of mtDNA.
Overall, this is a strong observational manuscript with meticulous attention placed on data collection and analysis. The conclusions drawn surrounding the role of MGME1 and linear deleted mtDNA lack additional experimental evidence. However, the conclusions drawn from the experiments presented can be adjusted if further experimental evidence cannot be provided to move forward with the current claim.

Response:

We have never been able to observe linear deleted mtDNA molecules in human cell lines (lacking mutations in MGME1) or in wild-type mice by using Southern blots or deep sequencing of mtDNA (see e.g., Matic et al., 2018). It is therefore not possible to study degradation of linear deleted mtDNA in a wild-type setting. In the revised manuscript, we provide additional data showing that the amounts of total mtDNA (full-length mtDNA + linear deleted mtDNA) and full-length mtDNA are significantly decreased in heart and kidney of MGME1 knockout animals (Fig 2C and D, Fig S3 A and B). Furthermore, we do not see any significant age-associated effect on the degree of mtDNA depletion (Fig 2C and D, Fig S3 A and B). These data provide additional support that mtDNA replication is impaired in mice lacking MGME1. The main conclusion we make based on the presented data is that MGME1 is not essential for the degradation of linear mtDNA fragments as degradation is still happening in its absence. Please also see relevant responses to reviewers 1 and 2.

**Have all data underlying the figures and results presented in the manuscript been provided?**
Large-scale datasets should be made available via a public repository as described in the *PLOS Genetics* [data availability policy](https://eur01.safelinks.protection.outlook.com/?url=http%3A%2F%2Fjournals.plos.org%2Fplosgenetics%2Fs%2Fdata-availability&data=04%7C01%7Cnils-goran.larsson%40ki.se%7Cc0d0302e0fb24b3361ec08d9d6b67657%7Cbff7eef1cf4b4f32be3da1dda043c05d%7C0%7C0%7C637776902308675824%7CUnknown%7CTWFpbGZsb3d8eyJWIjoiMC4wLjAwMDAiLCJQIjoiV2luMzIiLCJBTiI6Ik1haWwiLCJXVCI6Mn0%3D%7C1000&sdata=7X6fZRCI%2FFuGkUZmVBfqVjMqWDGl6NxEbGSWhWDkFAo%3D&reserved=0), and numerical data that underlies graphs or summary statistics should be provided in spreadsheet form as supporting information.

Reviewer #1: **No:** There are no examples of the Southern blots used for the quantification of the linear mtDNA fragments in the Supporting information.

Response:

We have now included the Southern blots used for quantification in the Supporting information section (Fig S2).

Reviewer #2: Yes

Reviewer #3: Yes
